# Assessment of Dysfunctional Cognitions in Binge-Eating Disorder: Factor Structure and Validity of the Mizes Anorectic Cognitions Questionnaire-Revised (MAC-R)

**DOI:** 10.3389/fpsyg.2017.00208

**Published:** 2017-02-16

**Authors:** Isabelle Carrard, Stephane Rothen, Maaike Kruseman, Yasser Khazaal

**Affiliations:** ^1^Department of Nutrition and Dietetics, School of Health Sciences, University of Applied Sciences and Arts Western Switzerland (HES-SO)Geneva, Switzerland; ^2^Department of Psychiatry, Geneva University HospitalsGeneva, Switzerland

**Keywords:** binge-eating disorder, cognition, factor analysis, statistical, questionnaire validity, severity

## Abstract

**Background:** Dysfunctional cognitions regarding weight and shape and their implications for self-esteem are considered core features of anorexia nervosa and bulimia nervosa. However, they have also been associated with the severity of binge eating disorder (BED). Therefore, they should be screened with appropriate instruments to tailor treatment to individual patient needs. The Mizes Anorectic Cognitions-Revised (MAC-R) is a self-report questionnaire that lists dysfunctional cognitions related to three hypothesized core beliefs typical of the psychopathology of eating disorders: weight and eating as the basis of approval from others; the belief that rigid self-control is fundamental to self-worth; and the rigidity of weight- and eating-regulation efforts.

**Objectives:** The goal of the study was to confirm the factor structure and to assess the validity of the MAC-R among a sample that met full-threshold and subthreshold criteria for BED.

**Methods:** We used data of women meeting full-threshold (*n* = 94) and subthreshold (*n* = 22) criteria for BED to conduct confirmatory factor analyses and to compute Spearman's correlations, in order to assess factorial, convergent, and discriminant validity.

**Results:** Two models having a structure of three factors with or without a total score proved to be acceptable. The MAC-R total score was correlated with questionnaires assessing dimensions related to eating disorder psychopathology, adding to the validity of the questionnaire.

**Conclusion:** These results were similar to those found in studies on the psychometric properties of the MAC among samples with anorexia nervosa or bulimia nervosa, encouraging the use of the MAC-R as a research or clinical tool in order to further document the core beliefs underlying BED.

## Introduction

After a “provisional” description in Appendix B of the 4th edition of the Diagnostic and Statistical Manual of Mental Disorders (DSM-IV; 1), the diagnosis of binge-eating disorder (BED) has acquired autonomy in the feeding and eating disorders section of the DSM-5, published in May 2013 (American Psychiatric Association, 2013). BED is characterized by recurrent episodes of binge eating without inappropriate compensatory behaviors, which are intended to prevent weight gain and are typical of bulimia nervosa (BN). In this latest edition of the DSM, the minimal frequency of binge-eating episodes and duration of the disorder have been matched to the criteria required for BN. Criteria for BED are mainly behavioral and require a certain level of distress caused by the behavior. However, they do not contain any criterion related to potential particularities of cognitive functioning, whereas BN and anorexia nervosa (AN) diagnoses include the excessive influence of shape and weight for self-esteem, which is considered a core characteristic of both of these disorders (Fairburn, 2008).

The possibility of including a criterion that would address dysfunctional cognitions in the BED diagnosis had been discussed prior to the release of the DSM-5 (Mond et al., 2007) and continues to be discussed (Harrison et al., 2015). As a matter of fact, the overvaluation (resulting in an overinvestment) of shape and weight in the construction of self-esteem has been observed among BED patients, albeit not all of them. Those with this specific characteristic also exhibited greater levels of psychopathology than BED patients without these preoccupations (Grilo et al., 2010). Moreover, overvaluation of shape and weight appeared to have a predictive validity regarding post-treatment levels of binge-eating frequency (Masheb and Grilo, 2008). For these reasons, Grilo et al. (2009) suggested that the presence of these dysfunctional cognitions could be used as a severity rating of BED rather than as a mandatory criterion for the diagnosis.

Dysfunctional cognitions in eating disorders were first conceptualized by Garner and Bemis (1982) and by Fairburn (1985), who emphasized the importance given by patients suffering from AN or BN to the self-control of eating and weight for the evaluation of their self-esteem. Vitousek and Hollon (1990) described eating disorders in terms of schemas underlying AN and BN dysfunctional cognitions and attitudes, schemas that are related to the self (the person being highly self-critical, judging himself or herself as being ineffective or inadequate), to weight (suffering from obesity or being overweight being rigidly associated with personal faults or flaws), and to the relationship between the weight and the self (a combination of the two previous schemas, leading to considering shape and weight as unique references for evaluating self-esteem). Schemas, or core beliefs, affect an individual's selection and interpretation of information, through biases of attention, memory, and judgment. In this way, cognitive schemas perpetuate the psychopathology (Vitousek and Hollon, 1990). Therefore, dysfunctional cognitions should be screened and addressed during therapy because they reveal how underlying core beliefs operate in the patient's functioning.

In order to investigate dysfunctional cognitions, we need tools that are validated and easy to administer. The Mizes Anorectic Cognitions Questionnaire (MAC) is one of the validated self-administered questionnaires assessing dysfunctional cognitions in eating disorders (Mizes and Christiano, 1995). The MAC development was specifically based on the tripartite model of schemas characteristic of AN and BN proposed by Garner and Bemis (1982), leading to a questionnaire that covers dysfunctional thoughts related to three dimensions: perception of weight and eating as the basis of approval from others; the belief that rigid self-control is fundamental to self-worth; and the rigidity of weight- and eating-regulation efforts (Mizes and Klesges, 1989).

The first version of the MAC contained a pool of 45 potential items developed by research assistants and selected by judges. This 45-item version was able to distinguish between populations of patients with BN and control participants (Mizes, 1988). The psychometric properties of the MAC were then extensively investigated (Mizes and Christiano, 1995). A principal component analysis carried out on the data provided by 205 college students produced a solution that selected 33 items divided into three factors (Mizes and Klesges, 1989). This 33-item version showed good internal consistency and a convergent validity with questionnaires assessing binge eating and body image distortion. The criterion validity was demonstrated with the MAC showing more sensitivity to subclinical differences in eating-related behavior and attitudes (Mizes, 1990). The construct validity was confirmed in a further study in which the MAC was completed by a sample of 100 female undergraduates together with questionnaires classically used to assess various aspects of eating disorders, such as the BULIT (Smith and Thelen, 1984) or the EAT-26 (Garner and Garfinkel, 1979), and other supposedly unrelated measures, such as social desirability, academic ability, or subscales assessing Type A dimensions (Mizes, 1991). All correlation coefficients for conceptually related measures were positive and statistically significant, whereas correlations with unrelated scales were not significant, providing more evidence for the validity of the MAC. Test-retest reliability was also demonstrated (Mizes, 1991). Moreover, in order to check whether the MAC was not a nonspecific evaluation of psychopathology, scores of patients with BN (*n* = 15) or AN (*n* = 8) or suffering from other psychiatric disorders (*N* = 11) were compared (Mizes, 1992). The criterion validity of the MAC was supported, the group with other psychiatric diagnoses obtaining largely lower scores on the MAC total score and subscales in comparison with the two groups with eating disorders, whose scores were similar.

To increase the MAC's psychometric properties, a shorter version of 24 items called the MAC-Revised (MAC-R) was developed (Mizes et al., 2000). A sample of 205 patients with AN, BN, or an eating disorder not otherwise specified (EDNOS) completed the original MAC, an addition of 24 new pilot items, and two other questionnaires to assess eating attitudes: the Eating Disorder Inventory (Garner et al., 1983) and the Restraint-Scale Revised (Gorman and Allison, 1995). A principal component analysis produced a solution that retained 24 items and three factors of equal length, with a high internal consistency for each subscale and the total score. The construct validity assessed by the mean of correlations with the two questionnaires on eating attitudes was good (Mizes et al., 2000). Contrary to the MAC, the MAC-R could distinguish the group of patients with the AN restrictive subtype from the group of patients with BN, the former obtaining lower scores on the MAC-R total score and two of the subscales (Mizes et al., 2000).

More recently, the MAC and the MAC-R were completed by 201 nonclinical adolescents to examine the differences of scores between gender and racially diverse samples (Peak et al., 2012). No differences were found across races, but females obtained higher scores of dysfunctional cognitions than males. Moreover, this study also confirmed similar internal consistency of the MAC and MAC-R subscales, as well as convergent validity with questionnaires assessing body satisfaction and fear of weight gain. As in a previous study (Mizes and Klesges, 1989), no differences were found between normal weight or overweight groups. However, a study by Osman et al. (2001) failed to confirm the factor structure of the MAC-R in a sample of 290 undergraduates. Instead, a brief 12-item version with three oblique factors was retained and called the BMAC (for brief version of the MAC-R). This new 12-item version should be tested and validated with a clinical population to decide its relevance.

In the present study, we used a French version of the MAC-R, which was adapted in the University Hospitals of Geneva, Switzerland. The English version of the MAC-R was obtained from Mizes, translated into French, and then tested. The translation that was finally obtained was judged accurate by a sworn translator. The French version of the MAC-R was used for the first time in an exploratory study by Volery et al. (2006), who compared the dysfunctional cognitions shown by samples of obese patients with (*n* = 18) and without (*n* = 11) BED and a control group with a normal weight (*n* = 13). The authors found that both groups of patients with obesity exhibited more dysfunctional cognitions than the control group (Volery et al., 2006). Two other studies involving clinical samples suffering from psychotic disorders also used the French adaption of the MAC-R (Khazaal et al., 2007, 2010). A decrease of the dysfunctional cognitions related to eating and weight following a cognitive behavioral intervention could be seen in 61 patients who had gained weight because of their treatment with antipsychotic drugs (Khazaal et al., 2007). However, again, the factor structure of the French MAC-R could not be confirmed with a sample of 125 patients with a diagnosis of schizophrenia or schizoaffective disorder (Khazaal et al., 2010). Confirmatory factor analyses highlighted that the shorter 12-item version proposed by Osman et al. (2001) provided a better fit to the data than the 24-item MAC-R, but the internal reliability of the scales obtained with the short 12-item version ranged from poor to acceptable.

The studies carried out until now have targeted students or clinical populations suffering from AN and BN, or other psychiatric disorders, but, to our knowledge, not BED *per se* because this diagnostic was not formally included in the DSM-IV (American Psychiatric Association, 1994). When it was developed, the MAC was designed to assess dysfunctional cognitions of all eating disorders, in spite of the reference to anorectic cognitions in its name (Mizes and Sloan, 1998; Mizes et al., 2000). In agreement with a transdiagnostic view of eating disorders, similar dysfunctional cognitions are core to the maintenance of the disorder in the full spectrum of the eating disorder psychopathology (Fairburn, 2008). Among the domains covered by the MAC-R, overinvestment of shape and weight has been judged relevant to evaluate in BED samples (Masheb and Grilo, 2008). Therefore, the goal of this study was to confirm the factor structure of the MAC-R among a sample that met full-threshold and subthreshold criteria for BED and to evaluate some aspects of its validity.

## Methods

All participants that provided data for the present analyses were included in studies on the efficacy of an Internet treatment for BED (Carrard et al., 2011a,b). The studies took place in the University Hospitals of Geneva (HUG), Switzerland, and were approved by the HUG Ethical Committee.

### Questionnaire and procedure

Participants were recruited partly in the community through an advertisement inviting people suffering from symptoms of BED to test a new form of treatment and partly in the service of the HUG, where the studies were hosted. To be included in the study, participants had to be women between 18 and 70 years old who were fluent in French, had average Internet skills, and met full-threshold or subthreshold diagnostic criteria for BED according to the DSM-5. They were excluded if they had recently attempted suicide or had undergone obesity surgery.

All participants completed a French version of the MAC-R questionnaire at baseline. The questionnaire contains 24 items that are rated on a 5-point Likert scale ranging from 1 “strongly disagree” to 5 “strongly agree,” with 10 reversed scores items. A total score as well as a score for each subscale can be computed: self-control as fundamental for self-esteem (SELF-CONTROL, 8 items), rigid weight regulation and fear of weight gain (RIGID WEIGHT REGULATION, 8 items) and weight and eating as the basis of approval from others (WEIGHT APPROVAL, 8 items).

The full-threshold or subthreshold diagnosis of BED stemmed from an interview that took place before inclusion in the Internet self-treatment program, to check inclusion and exclusion criteria. This interview, conducted by an experienced psychologist, was standardized with the Eating Disorder in Obesity (de Man Lapidoth et al., 2007), which rephrases questions from each BED criterion.

A series of other questionnaires assessing demographics, eating disorders, and psychological health was also completed by the participants. To assess construct validity, we correlated the MAC-R with participants' age and BMI and with the following three selected questionnaires.

The Eating-Disorder Evaluation-Questionnaire (EDE-Q) (Fairburn and Beglin, 2008) is a 28-item scale that includes four subscales assessing core dimensions of eating disorders: Restraint, Eating Concern, Shape Concern, and Weight Concern. A total score can be calculated that reveals a general level of eating disorder psychopathology. Participants with high scores on the EDE-Q should also obtain elevated scores on the MAC-R total score and subscales.

The Beck Depression Inventory (BDI) (Beck et al., 1996) is a 21-item scale that evaluates somatic and psychological signs of depression and gives a total score of severity. Because negative affect is correlated with more severe forms of BED psychopathology (Stice et al., 2001), it was hypothesized that higher scores on the BDI should be correlated with more dysfunctional cognitions on the MAC-R total score and subscales.

The Urgency, (lack of) Premeditation, (lack of) Perseverance, Sensation Seeking (UPPS) (Whiteside and Lynam, 2001) is a 51-item questionnaire that assesses four facets of impulsivity. The facet of Urgency, which is the tendency to act rashly in a condition of negative affect, has been found to be the most strongly correlated with bulimic symptoms (Fischer et al., 2008). Therefore, correlations between Urgency and the MAC-R total score and subscales should be found. On the other hand, the three other facets of the UPPS questionnaire should not be correlated with any MAC-R score.

### Population

Forty-two women were recruited in the hospital service and 74 in the community, resulting in a sample of 116 women, with a mean age of 38.5 years old [*SD* = 11.4, range = (21;70)] and a mean body mass index (BMI) of 31.4 [*SD* = 6.5, range = (19.4;49.7)]. Twenty-two participants (19%) reported a BMI lower or equal to 25 kg/m^2^, 25 (22%) reported a BMI from 25 to 30 kg/m^2^ (included), 34 (29%) reported a BMI from 30 to 35 kg/m^2^ (included), and 34 (29%) reported a BMI higher than 35 kg/m^2^.

The full-threshold criteria for BED were met by 81% (*n* = 94) of the participants, and the remaining 19% (*n* = 22) met subthreshold criteria. The patients with subthreshold criteria reported less than one binge-eating episode per week on average during the last 3 months, but the intensity of the disorder had varied between full-threshold and subthreshold criteria throughout their entire lives, and their suffering was significant enough to ask for treatment at the moment of the inclusion. The age when binge eating appeared was difficult for patients to remember and ranged from during childhood to 6 months ago.

The majority of the sample was employed at the time of the study, either full-time (*n* = 40, 34.5%) or part-time (*n* = 45, 38.8%). The rest was studying (*n* = 6, 5.2%), unemployed (*n* = 4, 3.4%), raising children at home (*n* = 12, 10.3%), or retired (*n* = 9, 7.7%). Most of the participants were living with a partner (*n* = 71, 61.2%) or with parents, children, or friends (*n* = 16, 13.8%), whereas 29 participants (*n* = 25%) were living alone. Almost half of the sample had university training (*n* = 55, 47.5%).

### Data analyses

#### Confirmatory factor analysis

In order to assess the validity of the factor structure, a confirmatory factor analysis (CFA) was conducted. Three models were tested. Model 1, with only one factor, was driven by the fact that the three postulated subscales correlated highly with the total score (the smallest being 0.76 for the SELF-CONTROL subscale) and that the Cronbach's alpha was very high (0.90) for the total score, suggesting a single main factor. Model 2 tested the previously postulated “three factors model” (Mizes et al., 2000) composed of SELF-CONTROL, WEIGHT APPROVAL, and RIGID WEIGHT REGULATION. Model 3, mathematically equivalent to Model 2, included a higher order factor model, which involved the three postulated subscales and a second order main factor representing the total score. As the items were not normally distributed, the unweighted least-square (ULS) method was chosen as the procedure for estimation, since it does not assume any distribution of the manifest variables (Bollen, 1989). Six pre-established criteria were selected as indicators of the goodness of fit to the data: (1) the goodness-of-fit index (GFI); (Jöreskog and Sörbom, 1996) > 0.90, (2) the adjusted goodness-of-fit index (AGFI); (Jöreskog and Sörbom, 1996) > 0.80, (3) the normed-fit index (NFI), (Bentler and Bonett, 1980) > 0.90, (4) the Tucker-Lewis index (TLI); (Tucker and Lewis, 1973) > 0.95, (5) the comparative fit index (CFI); (Bentler, 1990) > 0.95, and (6) the root mean square error of approximation (RMSEA), (Steiger and Lind, 1987) <0.06. The use and cutoff of the GFI and the AGFI were recommended by Cole (1987), the NFI by Bentler and Bonett (1980), and the RMSEA, TLI, and CFI by Hu and Bentler (1999).

In the framework of structural equation modeling the assessment of sample size is complicated and a clear answer does not exist, especially when using ULS. Nevertheless, to address this concern, two different strategies have been considered. First, a power analysis for the RMSEA using the tool developed by Preacher and Coffman (2006). Second, the stability of the tested models has been assed using a non-parametric bootstrap procedure (Efron, 1987).

The reliability of the subscales was assessed by using the Cronbach alpha coefficients (Cronbach and Meehl, 1955) as a measure of internal consistency. Again, due to the categorical nature of the Likert scale, Cronbach's alpha were computed based on the polychoric correlation matrix (Gadermann et al., 2012).

CFA were carried out with the software R 3.1.0 (R Core Team, 2014) using the *Lavaan* package (Rosseel, 2012), bootsrap were performed using the *boot* package (Canty and Ripley, 2016), power analysis using the webtool (http://www.quantpsy.org/rmsea/rmsea.htm), Cronbach's alphas were computed using the *psych* package (Revelle, 2014), and polychoric correlation matrices were calculated using the GPArotation package (Bernaards and Jennrich, 2005).

#### External validity

Taking into account that the distributions of the MAC-R items were not normal, Spearman's correlations were used to assess MAC-R construct validity when correlated with the EDE-Q, BDI, and UPPS questionnaires. Because of the number of correlations, a conservative level of *p* < 0.001 was used to decide significance.

To evaluate whether the MAC-R could discriminate between BED severity levels, Mann-Whitney U tests were computed to compare the MAC-R total and subscale scores of the group with full-threshold BED criteria with those of the group with BED subthreshold criteria.

## Results

### Confirmatory factor analysis

Since Model 2 and Model 3 were mathematically equivalent, fit indices for these two models were identical. Fit indices suggest that Models 2 and 3 were better than Model 1 (Table 1), that is that the three subscales fit the data better than one single factor. There is no standard and clearly validated procedure to statistically compare models when the method of estimation is ULS. Nevertheless, two methods have been performed. First a significance test based on the fitting function was done, which is equivalent to the well-known Chi-square test. The test showed that model 2 and 3 were better than model 1 (Fitting-function difference = 91.44, *df* = 3, *p* < 0.001). This test has to be taken with caution. Second, the difference in CFI has been carried out according to Cheung and Rensvold (2002). A change in the CFI greater or equal to 0.01 suggested a significant difference in fit, which is the case here (CFI in Model 1 = 0.91, CFI in Model 2 and 3 = 0.94). This procedure is usually done within the measurement invariance across group framework and again should be taken with caution. Nevertheless, raw comparisons as well as statistical testing suggest that Model 2 and 3 better fitted the data than Model 1.

**Table 1:** **Fit indices from unweighted least-square confirmatory factor analysis**.

	**Goodness-of-fit index**	**Adjusted goodness-of-fit index**	**Normed-fit index**	**Tucker-Lewis index**	**Comparative fit index**	**Root mean square error of of approximation (95% C.I.)**
Model 1	0.94	0.93	0.85	0.90	0.91	0.10 [0.09;0.11]
Model 2 or 3	0.95	0.94	0.87	0.93	0.94	0.08 [0.07;0.10]

According to the cutoff defined above, two out of six goodness-of-fit indices were considered excellent, whereas the NFI, the RMSEA, the CFI, and the TLI were slightly under the recommended value. Therefore, the construct validity of Models 2 and 3 were defined as acceptable. Moreover, Cronbach's alphas were good for the total score (0.86), WEIGHT APPROVAL (0.80) and RIGID WEIGHT REGULATION (0.80) and acceptable for the SELF-CONTROL subscale (0.76).

Factor loadings and correlations from the CFA of Model 2 and Model 3 are displayed in Table 2. The signs of the loadings were in accordance with the postulated scoring key since some items (e.g., item 11) must be reversed. This added to the construct validity of the questionnaire.

**Table 2:** **Results of unweighted least-square confirmatory factor analysis for Models 2 and 3: Factor loadings and correlations**.

**Latent variables: Models 2 and 3**	**Estimate**	**Standard error**
**SELF-CONTROL**		
item 1	1.00	
item 4	0.67^*^	0.13
item 9	0.51^*^	0.12
item 11	−1.46^*^	0.19
item 13	0.96^*^	0.15
item 18	−1.87^*^	0.23
item 20	−1.04^*^	0.16
item 23	−1.54^*^	0.20
**WEIGHT APPROVAL**		
item 3	1.00	
item 6	−0.43^*^	0.05
item 8	−0.66^*^	0.05
item 10	−0.83^*^	0.06
item 15	−0.64^*^	0.05
item 17	0.87^*^	0.06
item 21	−0.39^*^	0.05
item 24	−0.62^*^	0.05
**RIGID WEIGHT REGULATION**		
item 2	1.00	
item 5	1.19^*^	0.07
item 7	0.49^*^	0.05
item 12	1.08^*^	0.07
item 14	0.71^*^	0.05
item 16	0.58^*^	0.05
item 19	0.50^*^	0.05
item 22	0.88^*^	0.06
**MODEL 2: CORRELATIONS**		
Self-Control—Weight Approval	0.63^*^	
Self-Control—Rigid Weight Regulation	0.61^*^	
Weight Approval—Rigid Weight Regulation	0.74^*^	
**MODEL 3: FACTOR LOADINGS**		
**Main**		
Self-Control	1.00	
Weight Approval	2.70^*^	0.34
Rigid Weight Regulation	2.51^*^	0.33

* *p < 0.001*.

Regarding sample size assessment, as described in the Methods section, first a Power analysis was carried out. Alpha was set at 0.05, Null RMSEA at 0.05, Alternative RMSEA at 0.08, the number of degrees of freedom were 249. This provided a Power of 0.97 which suggest an adequate sample size. Second, a bootstrap procedure to assess model stability (Schumacker and Lomax, 2010) was performed. One thousand bootstrap samples have been generated and on each sample the three models have been fitted. A relative bias has been computed for every loading as follow: the original parameter minus the mean of this parameter across the 1000 samples divided by the original parameter, i.e.,

$$\text{relative bias}=\left|\frac{b_{i}-\bar{b_{i}^{*}}}{b_{i}}\right|$$

with:

- b_*i*_ the ith loading of the actual sample
- $\bar{b_{i}^{*}}$ the mean of the ith loading across the 1,000 samples.

Regarding Model 1, relative biases fell between 14 and 30% suggesting that the sample is either too small or that the model is inadequate. Regarding Models 2 and 3, two situations emerged. ForWEIGHT APPROVAL and RIGID WEIGHT REGULATION, relative biases fell between 0.02% and 2.45%, which is excellent, whereas for SELF-CONTROL they were between 19 and 62%. This suggests that the sample size is partially adequate but casts some doubt on the SELF-CONTROL subscale.

### External validity

Spearman's correlations between MAC-R, BMI, EDE-Q, BDI, and UPPS are displayed in Table 3.

**Table 3:** **Spearman's correlations between Mizes Anorectic Cognitions-Revised (MAC-R) and Eating Disorder Examination-Questionnaire (EDE-Q), Beck Depression Inventory (BDI), and Urgency, (lack of) Premeditation, (lack of) Perseverance, Sensation Seeking (UPPS) questionnaire**.

**Mizes Anorectic Cognitions-Revised (MAC-R)**	**Self-Control**	**Rigid Weight Regulation**	**Weight Approval**	**Total Score**
**EDE-Q**				
Restraint	0.399^*^	0.297	0.150	0.346^*^
Eating Concern	0.480^*^	0.295	0.387^*^	0.475^*^
Shape Concern	0.548^*^	0.466^*^	0.599^*^	0.673^*^
Weight Concern	0.593^*^	0.421^*^	0.559^*^	0.652^*^
Total Score	0.671^*^	0.483^*^	0.541^*^	0.696^*^
**BDI**				
Total Score	0.271	0.495^*^	0.393^*^	0.499^*^
**UPPS**				
Urgency	0.370^*^	0.191	0.332^*^	0.375^*^
Lack of Premeditation	−0.119	−0.217	0.106	−0.068
Lack of Perseverance	0.017	−0.070	0.142	0.070
Sensation Seeking	0.029	0.063	0.0183	0.133
**PARTICIPANTS' CHARACTERISTICS**				
Age	−0.295	−0.071	−0.348^*^	−0.277
BMI	−0.198	−0.105	−0.018	−0.131

* *sig, p < 0.001*.

Participants with higher scores on the MAC-R total score also obtained higher scores on the four subscales of the EDE-Q (Restraint, Eating Concern, Shape Concern, and Weight Concern) and the EDE-Q total score. The MAC-R total score was also positively associated with the BDI total score and with the UPPS facet of Urgency. No correlations were found between the MAC-R total score and the three other facets of the UPPS. This pattern was the one expected to provide evidence in favor of the convergent and discriminant validity of the MAC-R.

For the three MAC-R subscales, the results found were less uniform. The SELF-CONTROL subscale showed the expected pattern except that it was not correlated with the BDI total score. The WEIGHT APPROVAL subscale was not correlated with the EDE-Q Restraint subscale. The RIGID WEIGHT REGULATION subscale was not correlated with the EDE-Q Restraint and Eating Concern subscales, nor with the Urgency facet of the UPPS.

Finally, the BMI was not statistically associated with the MAC-R total score or subscales, but the participant's age was negatively associated with the MAC-R WEIGHT APPROVAL subscale, meaning that the older the participants were, the less they scored on this subscale.

### Criterion validity

Table 4 shows the medians, interquartile ranges, and minimum-maximum ranges obtained by the two groups of participants meeting BED full-threshold or subthreshold criteria. Higher scores were observed for the BED full-threshold criteria group, which is the more severe group, on the SELF-CONTROL and RIGID WEIGHT REGULATION subscales and the MAC-R total score. However, the null hypothesis presuming no differences between both groups could not be ruled out when Mann-Whitney U tests were computed.

**Table 4:** **Median, interquartile range (IQR), minimum-maximum (range), Mann-Whitney U test results for the three MAC-R subscales and total score, and the two groups' comparisons**.

**MAC-R**		**Subthreshold BED *n* = 23**	**Full-threshold BED *n* = 93**	**Group comparison**	**Total *n* = 116**
Self-control	Median	31.0	33.0		33.0
	IQR	7.0	6.0		6.8
	Range	20.0–39.0	19.0–40.0		19.0–40.0
	Mann-Whitney U			871.0	
	p			0.168	
Rigid weight regulation	Median	20.0	22.0		21.0
	IQR	6.0	10.0		9.8
	Range	11.0–32.0	9.0–39.0		9.0–39.0
	Mann-Whitney U			845.5	
	p			0.120	
Weight and approval	Median	21.0	21.0		21.0
	IQR	6.0	9.0		8.8
	Range	11.0–32.0	10.0–36.0		10.0–36.0
	Mann-Whitney U			992.5	
	p			0.593	
Total score	Median	71.0	75.0		74.0
	IQR	16.0	19.0		19.8
	Range	43.0–98.0	47.0–105.0		43.0–105.0
	Mann-Whitney U			858.0	
	p			0.143	

## Discussion

Similarly to the structure found with exploratory factor analyses among samples of AN, BN, and EDNOS (Mizes et al., 2000), a model including three factors proved to be acceptable for individuals with full-threshold and subthreshold criteria for BED. The two methods used to compare the models confirmed that the two models involving three factors better fitted the data than the model including one single factor, even if these results have to be taken with caution. Moreover, the MAC-R total score was correlated with the EDE-Q subscales and total score, which represent an evaluation of the severity of the eating disorder psychopathology. Participants with more signs of depression and more urgency also exhibited more dysfunctional cognitions, whereas correlations were not significant for impulsivity facets that were expected to be less associated with the MAC-R. These results speak in favor of using the MAC-R to examine BED dysfunctional cognitions, in agreement with the transdiagnostic view of the eating disorder psychopathology (Fairburn, 2008). However, it should also be noted that THE RIGID WEIGHT REGULATION subscale showed fewer correlations with dimensions that should have been associated. This can be explained by the fact that BED is characterized by moderate dietary restraint, less prevalent than in AN and BN (Stice et al., 2001). Further studies should determine whether this subscale can discriminate between BED and nonclinical individuals to judge its relevance for the assessment of BED psychopathology.

No statistically significant correlations were found between the participants' BMI and their MAC-R scores. This result is similar to that obtained with the nonclinical population of adolescents in studies that failed to discriminate between normal weight and overweight groups with the MAC and the MAC-R (Mizes and Klesges, 1989; Peak et al., 2012). Dysfunctional cognitions on eating and weight can occur in individuals at every weight. An association was found between age and the MAC-R WEIGHT APPROVAL subscale, showing that the older the participants, the less they valued weight and eating as a basis of approval by others. Until now, the studies carried out have included young participants because AN and BN mainly affect young persons, whereas BED can be found in a wider age range of individuals (American Psychiatric Association, 2013) with the same severity (Guerdjikova et al., 2012). This is the first time that such a result has emerged, and it should be confirmed.

The median obtained by the group meeting full-threshold criteria for BED was higher for the total score, the subscales assessing self-control as fundamental for self-esteem and rigid weight regulation and fear of weight gain, and for the MAC-R total score. However, these differences did not reach significance. This could be due to the size of the subthreshold group. Moreover, the study participants that did not meet the full criteria at the time of inclusion had a suffering great enough to make them ask for treatment, raising the question of the relevance of this subthreshold distinction in our case. The loosening of the criteria of binge-eating frequency between the DSM fourth and fifth editions shows how difficult it is to find the “right amount” of symptoms that indicates that the pathology has to be considered. Two studies have also recently shown that overvaluation of shape and weight was a more robust indicator of severity of BED than frequency of binge eating in a clinical (Grilo et al., 2015a) and in a community sample (Grilo et al., 2015b).

In the MAC-R validation study, individuals with AN obtained lower scores than those with BN on the MAC-R total score and on the two subscales designating self-control as fundamental for self-esteem and weight and eating as the basis for approval (Mizes et al., 2000). No difference was found regarding rigid weight regulation and fear of gaining weight between those with AN and those with BN. Further investigations should explore the specific pattern BED patients would obtain on these scales in comparison with the two other eating disorder diagnoses. For example, because of stigma that affects those who are overweight in Western society, a subgroup of BED patients, the majority of whom suffer from obesity or being overweight (Kessler et al., 2013), could display greater scores on the subscale reflecting specifically beliefs that weight and eating are the basis of approval from others, compared to patients with AN or BN. The MAC-R could also lead to the categorization of BED patients into various groups of severity according to their profile of dysfunctional cognitions and therefore might help tailor the treatment in a relevant way.

BED is indeed characterized by heterogeneity of the disorder, which moderates the treatment response. Cluster analyses led to the identification of BED sub-types involving either patterns with pure dietary restraint or with dietary restraint and high negative affect (Grilo et al., 2001; Carrard et al., 2012). Moreover, latent class analyses highlighted that according to more or less prevailing signs (such as binge- or over-eating frequency, shape, and weight concern, negative affect, or BMI), BED patients responded better to interpersonal psychotherapy or cognitive behavioral therapy (Sysko et al., 2010). This shows the importance of taking into account BED patients' characteristics in order to adapt treatment strategies.

For now, even if behavioral weight loss programs or psychoeducational interventions showed favorable effects on BED when associated with less severe psychopathology, specific interventions such as psychotherapies and particularly, cognitive, and behavioral therapy showed a superior efficacy, underlying the relevance of addressing the cognitive factors that maintain the disorder in addition to behavioral symptoms (Amianto et al., 2015).

Cognitive and behavioral therapy used for BED treatment has been modeled on the CBT treatment first designed for BN (Iacovino et al., 2012). In the case of differences observed on the MAC-R between BN and BED, the core psychopathology of BED could be targeted more specifically in an adjusted model. Finally, beyond core beliefs that are focused on the necessity of eating and weight control and their importance for self-esteem, other schemas were found to be at stake for individuals with eating disorders, such as the belief that emotional expression has aversive consequences for BED particularly (Waller et al., 2000). An examination of various kinds of core beliefs that could differentiate the eating disorders from one another could be undertaken in further research and lead to the development and adjunction of other subscales.

Among the limitations of the study, the results of the sample size estimation carried out with the boostrap procedure indicated that the solution was potentially unstable. It means that the validity of the three-factor solution obtained with this study CFA should be confirmed in a larger sample. Then, the data used to assess the MAC-R psychometric properties were not primarily collected for that purpose. The use of a sample constituted exclusively of women to validate the factor structure of the MAC-R among a population with BED symptoms is also a limitation of this study. In fact, the diagnosis of BED is almost equally represented among men and women, contrary to what can be observed among patients with BN and AN (Kessler et al., 2013). Therefore, the factor structure should be verified with a sample including both genders. The inclusion of men in samples subject to factor validation of the MAC-R questionnaire led to the shortening of the scale to the 12-item BMAC in two studies with undergraduates (Osman et al., 2001) as well as with patients with psychosis (Khazaal et al., 2010). A further validation with BED patients of both genders should be conducted to decide the more appropriate version of the MAC for this disorder.

## Author contributions

IC and YK conceptualized the study. IC collected the data. SR analyzed the data and wrote the statistical section of the manuscript. IC, SR, MK, and YK contributed to drafting the manuscript and agree to be accountable for the content of the work.

## Funding

The data used in this study were collected in two studies partially supported by the Hans Wilsdorf Foundation and funding from the Marie Curie Research Training Network INTACT (Individually Tailored Stepped Care for Women with Eating Disorders; MRTN-CT-2006-035988).

### Conflict of interest statement

The authors declare that the research was conducted in the absence of any commercial or financial relationships that could be construed as a potential conflict of interest.
